# The α7 Nicotinic Acetylcholine Receptor Agonist GTS-21 Improves Bacterial Clearance *via* Regulation of Monocyte Recruitment and Activity in Polymicrobial Septic Peritonitis

**DOI:** 10.3389/fimmu.2022.839290

**Published:** 2022-03-04

**Authors:** Jian-nan Hu, Ying Liu, Shu-chang Liu, Teng Zhang, Gui-bing Chen, Jie Zhao, Tao Ma

**Affiliations:** ^1^Department of General Surgery, Tianjin Medical University General Hospital, Tianjin, China; ^2^Department of Integrated Traditional Chinese and Western Medicine, Tianjin First Central Hospital, Tianjin, China

**Keywords:** GTS-21, cholinergic anti-inflammatory pathway, monocytes, bacterial clearance, septic peritonitis

## Abstract

The cholinergic anti-inflammatory pathway has been identified as an effective pathway to modify inflammatory responses. Here, we verified that delayed administration with a selective α7nAChR agonist GTS-21 enables a more efficient elimination of the offending pathogens, diminished inflammatory response and organ injury, and improved survival rates in the polymicrobial septic peritonitis model. We illustrated that the improved bacterial clearance upon GTS-21 stimulation was accompanied by enhanced recruitment of monocytes into the peritoneal cavity and simultaneously increased phagocytic activity and iNOS expression of these recruited monocytes. Mechanically, splenectomy prior to administration of GTS-21 attenuated the recruitment of monocytes into the peritoneal cavity and abolished the protective benefits of GTS-21 treatment. Meanwhile, GTS-21 administration accelerates the deployment of splenic monocytes during septic peritonitis. Collectively, these data suggested that appropriate selective pharmacological α7nAChR activation promotes monocytes trafficking in a spleen-dependent manner and upregulates the antibacterial activity of recruited monocytes during septic peritonitis, which may be utilized as a promising therapeutic modality for patients suffering from septic peritonitis.

## Introduction

Sepsis is a serious global health problem (1). In 2017, an estimated 48·9 million (95% uncertainty interval 38·9–62·9) incident cases of sepsis were recorded worldwide and 11·0 million (10·1–12·0) sepsis-related deaths were reported, representing 19·7% (18·2–21·4) of all global deaths (2). Despite technical advances in intensive care and the development of the novel generation of antibiotics, the mortality rate remains extremely high, ranging from 30-70% depending on the underlying cause and the organs affected (3). Thus, there exists an urgent need to find effective approaches for treating sepsis. Over the past two decades, accumulating evidence describes the interactions between the nervous system and immunity which culminated in the recognition of the intriguing neuroimmune regulation. These advances about the prompt and precise immune-regulation *via* the neuroimmune mechanism provided a basis for investigating new therapeutic interventions for combatting inflammation and infectious disorders (4–6).

Studies have dissected that the cholinergic nervous system reflexively monitors and modifies the inflammatory responses (7, 8). This so-called cholinergic anti-inflammatory pathway (CAP) is initiated in brain stem nuclei and exerts its effects *via* the vagus nerve by releasing acetylcholine (ACh) (9). Surgical division of the vagus nerve significantly exaggerated inflammation and increased susceptibility to infection (10). Subsequent work identified that the α7 subunit of nicotinic ACh receptors (α7nAChR) are essential for immune-modulating effects of the CAP signaling (11, 12). Accordingly, activation *via* vagus nerve stimulation or pharmacological α7nAChR agonists has been proved to regulate pro-inflammatory cytokine production and prevent lethal tissue injury in multiple models of inflammation and sepsis (13–15).

In the context of sepsis, the clinical applicability of vagal stimulation is limited by the invasive procedure required for direct nerve stimulation, then, special attention is given to alternative approaches using pharmacological tools mimicking the vagal anti-inflammatory signaling. As a selective α7nAChR agonist, the influences of GTS-21 in the regulation of inflammatory responses and immune function have drawn attention because GTS-21 treatment inhibits the synthesis and release of pro-inflammatory cytokines in macrophages and splenocytes. In addition, treatment with GTS-21 enhanced the differentiation of the naïve CD4^+^ T cells into Tregs and effector T cells (16, 17). Furthermore, in the present study, we proved that delayed administration with a selective α7nAChR agonist GTS-21 enables a more efficient elimination of the offending pathogen, diminished inflammatory response and organ injury, and improved survival rates in the CLP-induced polymicrobial sepsis model. The improved bacterial clearance correlates with enhanced recruitment of monocytes to the peritoneal cavity and increased functional activities of these monocytes. And we demonstrated that administration with GTS-21 accelerates the deployment of splenic monocytes during septic peritonitis, moreover, splenectomy eliminated the enhanced monocyte recruitment and survival benefit upon GTS-21 administration, suggesting that monocytes recruited to the peritoneal cavity originate from the spleen. Then, our findings identified the cholinergic modulation on monocytes in a spleen-dependent manner as a key participant in the innate immune response during bacterial sepsis and may have important implications for providing new therapeutic options for patients predisposed to or suffering from sepsis.

## Materials and Methods

### Animals

Male C57BL/6 mice (25-28g) were from Vital River Laboratory. Animals were housed in standard conditions with free access to food and water. All experimental manipulations were undertaken under the National Institute of Health Guide for the Care and Use of Laboratory Animals, with the approval of the Scientific Investigation Board of Tianjin Medical University.

### Cecal Ligation and Puncture Surgery

Septic peritonitis was induced by CLP as described previously (18, 19). In brief, mice were anesthetized with isoflurane inhalation. The cecum (0.5cm) was ligated and punctured once with a 20-gauge needle. A small piece of feces was extruded from the hole and the cecum was placed back into the abdominal cavity. Immediately after surgery, mice were administered with 1 mL sterile saline.

### GTS-21 Treatment

Groups of mice were treated with GTS-21 (provided by Abcam, 4 mg/kg) or vehicle (sterile saline) intraperitoneally 12h after CLP procedure and repeated every 12h respectively. At indicated time points, animals were sacrificed, and blood, peritoneal lavage fluid (PLF), and spleen, lung, and liver samples were collected for further evaluation. For survival analysis, mice were treated with GTS-21 or vehicle beginning 12h after CLP procedure and continued twice daily for three consecutive days. Survival was routinely evaluated for 7 days.

### Splenectomy

Mice were anesthetized with isoflurane inhalation. A midline laparotomy incision was made to expose and mobilize the spleen. And after splenic vessel ligation, the spleen was removed.

### Peritoneal Cells Isolation

The peritoneal cavity was injected with 5 mL of ice-cold PBS and massaged gently, then the PLF was extracted and washed twice with PBS by centrifugation at 4˚C at 300×g for 10 minutes. The resulting pellet was suspended in 1ml ice-cold PBS.

### Splenocytes Isolation

Mouse spleens were collected in ice-cold PBS and mechanically disrupted to obtain splenocytes. RBCs were lysed with RBC Lysis Buffer at room temperature and washed with PBS. Splenocytes were passage through an additional 40 μm filter, counted, and re-suspended in ice-cold PBS for further analysis.

### Flow Cytometry

Cells were blocked for Fc receptors with anti-mouse CD16/32 antibodies. For surface staining, cells were stained on ice for 30 mins with the following fluorescent conjugated antibodies: fluorescein isothiocyanate (FITC)–anti-Ly6G (1A8, Biolegend), peridinin-Chlorophyll-Protein Complex (PerCP)-anti-Ly6C Abs (HK1.4, Biolegend), phycoerythrin (PE)-anti-CD11b (M1/70, Biolegend) or allophycocyanin (APC)–anti-CD11b (M1/70, Biolegend). Staining for intracellular iNOS with APC–anti-iNOS (CXNFT, ThermoFisher scientific) was performed after fixation and permeabilization of the cells with a Cytofix/cytoperm kit (BD). Neutrophils were identified as CD11b+Ly6G+, and monocytes were identified as CD11b+Ly6G-Ly6C+ (**Supplementary Figures 1**–**4**). For *in vitro* phagocytosis assays, peritoneal cells were incubated with PE-labeled Escherichia coli (Abcam) for 2 hours, then these cells were recollected and labeled with surface markers CD11b, Ly6G, and Ly6C to identify monocytes. For ROS detection, peritoneal cells were incubated in a complete medium containing 5μM/ml ROS Brite™ (AAT Bioquest) for 30 minutes 37℃and then was stained with surface markers CD11b, Ly6G, and Ly6C to identify monocytes. Flow cytometry analysis was performed using the BD Accuir C6 Plus cell analyzer (BD Biosciences).

### Confocal Microscopy

Spleens were harvested and deposited in optimum cutting temperature compound, flash-frozen in liquid nitrogen. 5 μm-thick sections of the spleen were obtained and fixed in pre-cooled acetone for 20 min, blocked with 2% bovine serum albumin (BSA) for 30 min, and stained overnight at 4°C with Alexa Flour 660-anti-CD11b antibody (M1/70, Thermo Fisher Scientific). Tissue sections were washed and stained with a goat anti-rat IgG H&L preadsorbed fluorescent conjugated secondary antibody for 2 hours at room temperature. Sections were then washed three times in PBS, incubated with 4’,6-diamidino-2-phenylindole (DAPI) for 5 min before a final wash in PBS, and mounted with Prolong Gold antifade reagent (Invitrogen). Stained sections were imaged on a Zeiss LSM 800 confocal microscope with a Hamamatsu ORCA–ER CCD camera, data were analyzed by Zen software.

### Assessment of Cytokine Levels

Plasma and PLF were collected at indicated time points and analyzed using tumor necrosis factor (TNF) and interleukin 6 (IL-6) enzyme-linked immunosorbent assay (ELISA) kits (DAKEWE).

### Histopathologic Evaluation

Lung and liver samples were obtained at 30h after the CLP procedure. The collected samples were fixed in 4% paraformaldehyde, paraffin-embedded, and stained with hematoxylin and eosin(H&E). As previously described (20, 21), histological characteristics were assessed and scored under a light microscope, and the sum scores were calculated to determine injury scores.

### Bacterial Culture

PLF and blood samples for bacterial culture were obtained at indicated time pinots after CLP. These specimens were subjected to serially 10-fold dilution and cultured on Trypticase Soy Agar plates with 5% sheep blood at 37˚C for 24h. The numbers of bacteria colonies were calculated and expressed as Colony-forming units (CFUs) per milliliter.

### Statistical Analysis

All data were analyzed using GraphPad Prism software (GraphPad software). Except for CFUs, all values are expressed as mean ± SEM. Data were analyzed using a 2-tailed t-test. Differences among groups in bacterial CFU were compared using the nonparametric Mann–Whitney U test. Survival curves were analyzed using the log-rank test. A p-value of <0.05 was accepted as statistically significant.

## Results

### GTS-21 Mitigates Inflammatory Response, Alleviates Histopathology Damages, and Improves Survival Rates in Septic Peritonitis

**Figure 1:**
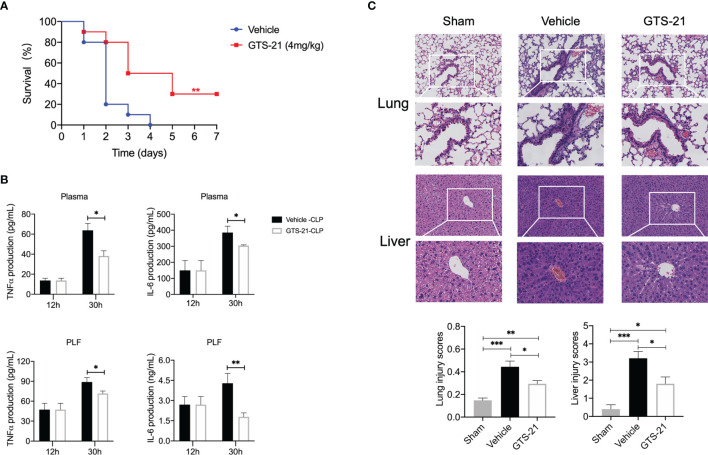
GTS-21 treatment suppresses local and systemic cytokines, alleviates histopathology damage, and improves survival rates during CLP-induced septic peritonitis. Mice were subjected to CLP, and GTS-21 or vehicle was injected intraperitoneally 12h after CLP procedure and repeated every 12h for three consecutive days. **(A)** The survival rates were evaluated for consecutive 7 days after CLP (n=10 mice/group). **(B)** PLF and blood samples were collected (*n*=6~8 mice/group) at an indicated time point, and the levels of TNF-α and IL-6 were measured using ELISA kits. **(C)** Lung and liver samples were obtained 30h after CLP (*n*=5 mice/group) and stained for H&E histopathology (original magnification, × 100 and ×200). The slides were histopathologically evaluated as described in Materials and Methods. All values represent means ± SEM. *p < 0.05; **p < 0.01; ***p < 0.001 for GTS-21 *vs* vehicle.

Considering its devoid of nicotinic toxicity, side effects, and well tolerance in healthy volunteers (22, 23), GTS-21, a selective α7nAChR agonist, was chosen in the present study. Given previous reports and our preliminary data showing that pretreatment or simultaneous usage with cholinergic agonists impaired innate host defense (15), mice were given either GTS-21 or vehicle saline (control group) intraperitoneally every 12h for consecutive 3 days, starting at 12h after CLP procedure. Consequently, this treatment significantly improved survival compared with vehicle-treated controls (30% *vs* 0%, **Figure 1A**). As expected, both PLF and plasma levels of TNFα and IL-6 were increased in the setting of septic peritonitis, which was inhibited significantly by GTS-21(**Figure 1B**), In addition, mice subjected to CLP showed histopathological evidence of organ injuries, including thickening of the alveolar septa and interstitial edema in lung sections, and swelling of hepatocytes and massive infiltration of neutrophils in liver sections, and GTS-21 treated mice showed a marked reduction in these septic-related organ injuries (**Figure 1C**).

### GTS-21 Improves Bacterial Clearance and Enhances Monocytes Recruitment in Septic Peritonitis

**Figure 2:**
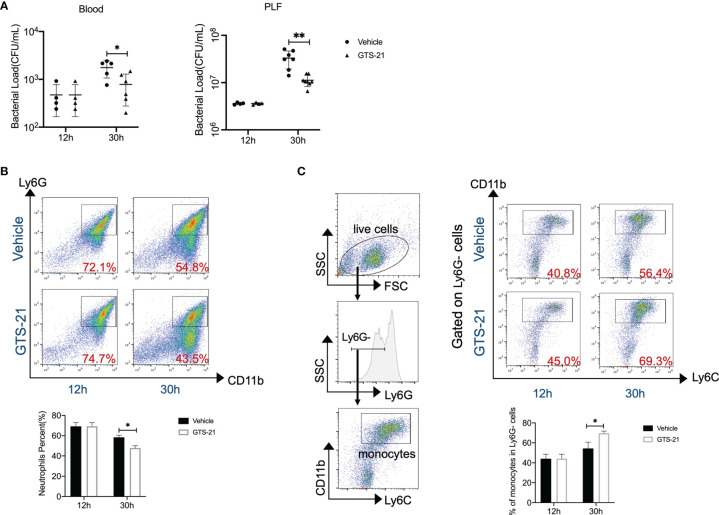
GTS-21 treatment improves bacterial clearance and enhances monocytes’ recruitment during CLP-induced septic peritonitis. Mice were subjected to CLP, and GTS-21 or vehicle was injected intraperitoneally 12h after CLP procedure and repeated every 12h. Then, GTS-21 or vehicle-treated mice were sacrificed at 12 and 30h after the CLP procedure. **(A)** PLF and peripheral blood were collected (*n*=6~8 mice/group) and bacterial CFUs were determined. The horizontal bar represents the mean for each group. Symbols represent individual mice. **(B, C)** Peritoneal cells were harvested (*n*=6~8 mice/group) and stained for neutrophils marker (CD11b+Ly6G+) or monocytes markers (CD11b+Ly6G-Ly6C+) respectively. Percentages of neutrophils or monocytes were measured by flow cytometry. Results are expressed as mean ± SEM. *p < 0.05; **p < 0.01 for GTS-21 *vs* vehicle.

CLP is regarded as the most representative animal model of human polymicrobial sepsis. Together with systemic inflammatory responses, the rapid spread of bacteria is one of the hallmarks in the pathophysiology of this model. Then, aiming to evaluate the consequences of GTS-21 treatment on bacterial clearance, bacterial loads in the PLF and blood were determined at indicated time points. Eventually, the PLF and blood CFU counts were noticeably lower in GTS-21-treated mice as compared with vehicle-treated control mice (**Figure 2A**). Mechanically, encountering pathogens, phagocytes including neutrophils and monocytes are recruited rapidly to abdominal infectious sites and are responsible for the clearance of microbial pathogens. While analyzing the recruitment of neutrophils, we found that percentages of neutrophils in the peritoneal cavity were lower at 30h after CLP in GTS-21 treated mice as compared with vehicle-treated mice (**Figure 2B**). Whereas, the proportion of CD11b+Ly6G-Ly6C+ monocytes in the peritoneal cavity was higher 30h after CLP procedure in GTS-21 treated mice when compared with control mice (**Figure 2C**), highlighting that it was monocytes but not neutrophils which is pivotal in improved antibacterial defense upon GTS-21 administration.

### GTS-21 Increases Peritoneal Monocytes Functions in Septic Peritonitis

**Figure 3:**
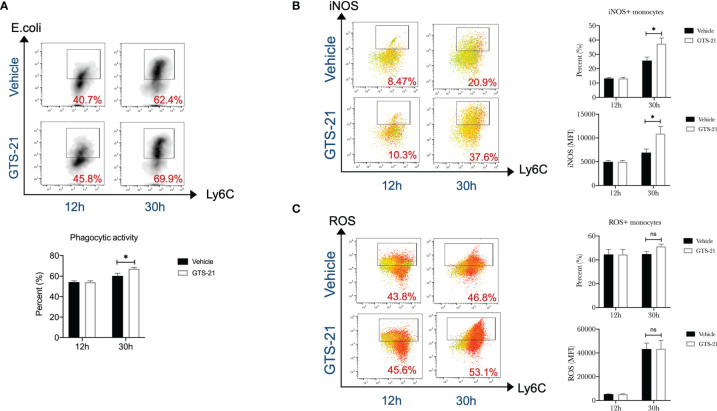
GTS-21 treatment increases peritoneal monocytes functions during CLP-induced septic peritonitis. Mice were subjected to CLP, and GTS-21 or vehicle was injected intraperitoneally 12h after CLP procedure and repeated every 12h. At indicated intervals, peritoneal cells were harvested (*n*=6~8 mice/group). **(A)** After isolation, peritoneal cells were incubated with PE-labeled Escherichia coli for 2h, then labeled with monocytes markers (CD11b+Ly6G-Ly6C+), phagocytic activity was evaluated by measuring the uptake of PE-labeled Escherichia coli by flow cytometry as described in Materials and Methods. **(B)** Then, after staining for monocytes markers (CD11b+Ly6G-Ly6C+), cells were permeabilized and assessed for intracellular expression of iNOS by flow cytometry as described in Materials and Methods. **(C)** After incubating with complete media containing 5μM/ml ROS Brite™ for 30 minutes, monocytes were determined by cell surface markers (CD11b+Ly6G-Ly6C+), ROS expression was determined by flow cytometry. Results are expressed as mean ± SEM. No statistical difference (ns, P>0.05) and *p < 0.05 for GTS-21 vs vehicle.

Monocytes are a subset of white blood cells and are trafficked to infectious sites, where they contribute to host immune defense against microbial pathogens (24). To effectively eliminate pathogens, monocytes are required to phagocytize the bacteria and destroy them after engulfment by producing reactive nitrogen species and reactive oxygen species (ROS), etc. Then, the phagocytic activity, iNOS expression, and ROS production of monocytes were qualified. We observed that peritoneal monocytes from septic mice treated with GTS-21 had enhanced phagocytic capacity when compared with those from vehicle-treated mice, as demonstrated by flow cytometric analysis of ingested PE-labeled Escherichia coli (**Figure 3A**). Similarly, monocytes isolated from GTS-21 treated mice had a significant increase in iNOS expression than those from vehicle-treated mice (**Figure 3B**). Though, GTS-21 treatment had no significant influence on monocytic ROS production (**Figure 3C**). Our data recommended that, together with increased recruitment, enhanced activity of these recruited monocytes may be responsible for improved bacterial clearance and improved survival implemented by GTS-21 treatment.

### The Spleen Is Required for the Protective Response of GTS-21 in the Treatment of Septic Peritonitis

**Figure 4:**
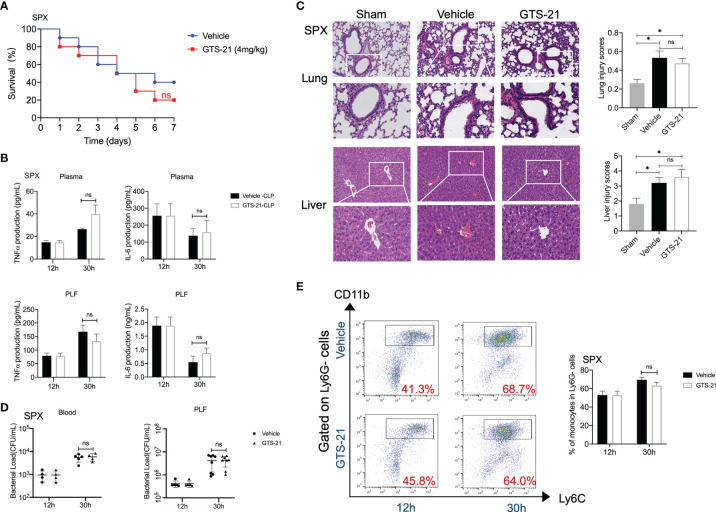
The spleen is required for the protective response of GTS-21 in the treatment of septic mice. Splenectomies were performed and 1 week later, splenectomized mice were subjected to CLP procedure. Then, GTS-21 or vehicle treatment began 12h after CLP and was administrated twice a day for 3 days. **(A)** To determine survival, mice were monitored for 7 consecutive days (*n*=10 mice/group). **(B)** At indicated time intervals, PLF and blood samples were collected (*n*=6~8 mice/group), PLF and plasma TNFα and IL-6 levels were measured using ELISA kits. **(C)** Pulmonary and liver samples were obtained (n=5 mice/group) and stained for H&E histopathology (original magnification, × 100 and ×200). The slides were histopathologically evaluated as described in Materials and Methods. **(D)** At indicated time point, peritoneal and serum bacterial loads in GTS-21 or vehicle-treated mice were quantified (n=6~8 mice/group). The horizontal bar represents the mean for each group. Symbols represent individual mice. **(E)** Splenectomized mice treated with GTS-21 or vehicle were sacrificed 30h after CLP, peritoneal cells were collected (n=6~8 mice/group) and stained for monocytes markers (CD11b+Ly6G-Ly6C+). Percentages of monocytes were measured by flow cytometry. Results are expressed as mean ± SEM. No statistical difference (ns, P>0.05) and *p < 0.05 for GTS-21 vs vehicle.

The critical functional significance of the spleen in the CAP signal has been well characterized (25). Then, to determine the role of the spleen in GTS-21-induced protective effects, we subjected mice to splenectomy or sham surgery before the induction of polymicrobial septic peritonitis. In contrast to the protective effects of GTS-21 stimulation in intact animals, splenectomy abolished the survival benefit of selective α7nAChR activation (**Figure 4A**). Accordingly, there were no differences in plasma or PLF levels of TNFα and IL-6 between GTS-21 and vehicle-treated mice (**Figure 4B**). Furthermore, histopathological examination of the lung and liver sections showed that there were no significant differences in organ injuries between GTS-21 treated or vehicle-treated splenectomized mice (**Figure 4C**). More importantly. we showed that splenectomy before GTS-21 administration fails to improve bacterial clearance, as evidenced by no significant differences in PLF and serum bacterial loads between GTS-21 and vehicle-treated mice (**Figure 4D**). Meanwhile, treatment with GTS-21 failed to enhance the recruitment of monocytes into the peritoneal cavity in splenectomized mice, because the percentages of peritoneal monocytes were similar in GTS-21 and vehicle-treated splenectomized mice (**Figure 4E**). Together, these results indicated that the spleen is required for the protective effects of GTS-21 in septic peritonitis.

### GTS-21 Enhances the Egress of Monocytes From the Spleen in Septic Peritonitis

**Figure 5:**
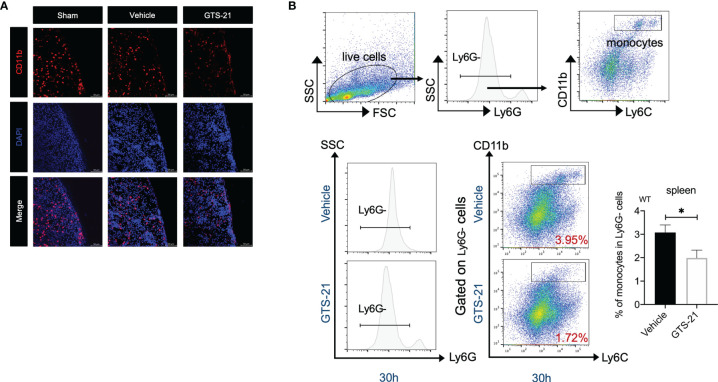
The spleen is required for improved bacterial clearance and enhanced monocytes recruitment in the peritoneal cavity during septic peritonitis. GTS-21 or vehicle was injected intraperitoneally 12h after CLP procedure and repeated every 12h. At indicated intervals, spleen samples were obtained from sham-operated, vehicle or GTS-21 treated mice 30h post-operation (n=5 mice/group) and were visualized with confocal microscopy as described in Materials and Methods. Representative images of spleen sections immunostained for CD11b are shown in **(A)**. **(B)** Spleens were harvested from vehicle or GTS-21 treated mice 30h after CLP, and cell suspensions were prepared (n=8 mice/group) and stained for monocytes markers (CD11b+Ly6G-Ly6C+). Percentages of monocytes were measured by flow cytometry. Results are expressed as mean ± SEM. *p < 0.05 for GTS-21 vs vehicle.

Recently, the spleen has been verified to contain a population of bona fide undifferentiated monocytes that could be deployed in response to injury or inflammation (26, 27). Indeed, as shown in **Figure 5A**, by using confocal microscopy, we detected dense populations of CD11b+ monocytes in the splenic subcapsular red pulp in sham-operated mice, and there was a significant decrease in CD11b+ cells in splenic sections after the CLP procedure, indicating that monocytes locate in the spleen are recruited to the peritoneum during abdominal infection. More importantly, even fewer CD11b+ cells were observed in the spleen of GTS-21 treated mice, highlighting that GTS-21 treatment leads to more robust monocytes egress from the spleen. Correspondingly, by using flow cytometry, we observed a significant decrease in the percentages of splenic monocytes in septic mice with GTS-21 treatment when compared with vehicle-treated mice (**Figure 5B**). Collectively, these data suggest that selective α7nAChR activation may serve as a modulatory signal for the deployment of splenic monocytes during septic peritonitis.

## Discussion

Recent researches bridging neuroscience and immunology have identified neural pathways that regulate immunity and inflammation and facilitated important advances for translational development (28, 29). Based on these advances, electrical stimulation of vagus neurons or pharmacological methods activating CAP is emerging as a promising therapeutic strategy in modulating and responding to inflammation and infection. Tracy and co-workers have studied the effect of electrical vagus nerve stimulation and chemical stimulation of α7 cholinergic receptors with either nicotine or selective α7nAChR agonists on the outcome of polymicrobial sepsis induced by the CLP procedure (13, 14). These studies consistently illustrated that activation of the CAP could reduce systemic inflammation and improve survival in these models. Correspondingly, blockade of the CAP with α7 cholinergic receptor antagonist contributes to the immune impairment in sepsis (30, 31). In line with these investigations, our current study confirmed the therapeutic potentials of chemical vagus nerve manipulation by GTS-21, even in the absence of antibiotic therapy. In this respect, it should be noted that some pieces of evidence argued that the protective effect of nicotinic agonist in CLP-induced sepsis may be the result of combined administration of antimicrobial drugs (32), and some other studies showed that nicotine pre-treatment would impair the innate host response, resulting in increased bacterial loads and worsen survival rates (33, 34). We postulated that the apparent discrepancy between the current study and earlier studies may be related to the differences in the time point of the intervention. In our set of experiments, GTS-21 was given at the time point of 12 hours after CLP and repeated every 12 hours for three consecutive days. We all know that, when encountering pathogens, an adequate inflammatory reaction is of paramount importance in shaping the host response to combat infection. Therefore, the activation of the CAP early in the pathogenesis of sepsis may hamper eliciting the appropriate immune response to fight against pathogens. However, in our set of experiments, delayed administration of this selective α7nAChR motivation would not interfere with the early production of sufficient inflammatory responses but facilitate the subsequent bacterial clearance as evidenced by lower bacterial burdens in both PLF and blood.

Classically, phagocytes, including neutrophils and monocytes, perform antimicrobial functions by phagocytosis, cytolysis *via* ROS and release, generation of antimicrobial peptides, etc. Upon infectious challenge, neutrophils and monocytes will be rapidly recruited from the vascular compartment to sites of infection, which is essential for successful bacterial containment and eradication. Then, aiming to elucidate the underlying mechanisms, the recruitment of neutrophils and monocytes to the peritoneal cavity was quantified after the induction of abdominal polymicrobial sepsis treated with or without GTS-21. Consistent with previous studies, we observed that treatment with GTS-21 inhibited neutrophils recruitment to the peritoneal cavity following CLP procedure, indicating that neutrophils are indispensable for improved microbial clearance in the setting of our experiments. However, we did observe that the percentages of monocytes were significantly higher in the peritoneal cavity of mice submitted to GTS-21 administration compared with control mice. Monocytes possess similar antimicrobial functions as neutrophils and are divided into two subsets based on different surface markers and phenotypic characteristics. Among these two subsets, CD11b^+^ Ly6C^high^ inflammatory monocytes will be recruited early from the bone marrow to sites of inflammation and actively participate in the phagocytosis of bacteria (35, 36). In line with our observations, Ocuin et al. demonstrated that neutrophils are not essential for survival in the CLP model, whereas simultaneous depletion of monocytes and neutrophils reduced survival significantly (37). Besides, Wang et al. proposed that monocytes are activated during abdominal sepsis and play a crucial role in the regulation of both systemic coagulation and inflammation (38). Even further, our data illustrated that GTS-21 treatment increased functional activities of monocytes within the peritoneum, as evidenced by the expression of iNOS and assessment of phagocytic capacity. Taken together, we postulated that it was the enhanced recruitment and functional activity of monocytes in the peritoneal cavity upon GTS-21 stimulation that was responsible for effective bacterial clearance and improved survival in our sets of experiments.

The spleen is a major secondary lymphoid tissue that contains lymphocytes in the white pulp and myeloid lineage cells (e.g., monocytes, macrophages, and dendritic cells) in the red pulp. Pieces of evidence have supported a critical role for this organ in the vagus nerve anti-inflammatory circuit. And in our data, we firstly demonstrated that the spleen is also required for the effective bacterial eradication during abdominal infection upon the CAP activation because splenectomy abolished the survival benefit of GTS-21 treatment. Mechanically, splenectomy before administration of GTS-21 attenuated the recruitment of monocytes into the peritoneal cavity. Recently, researchers have suggested that the spleen could serve as a site for storage and rapid deployment of monocytes and identifies splenic monocytes as a resource that the host exploits in response to inflammatory or infectious insults (26, 27, 39). Here, as shown in the present study, we detected obvious CD11b positive monocytes in the subcapsular red pulp of the spleen in sham-operated mice. After the CLP procedure, there was a significant decrease in CD11b+ cells in spleen sections, suggesting that these CD11b+ monocytes in the spleen are motivated to control intra-abdominal infection. And importantly, we demonstrated that this process of the deployment of splenic monocytes was enhanced by stimulation with GTS-21. Meanwhile, percentages of splenic monocytes decreased significantly in GTS-21 treated septic mice, as shown by flow cytometry analysis. Thus, it appears that, in addition to its classical action on modifying proinflammatory cytokine production, selective pharmacological α7nAChR activation accelerates the motivation of splenic monocytes into peritoneal infectious foci and then lead to optimized bacterial clearance and improved survival in this septic peritonitis.

Collectively, we demonstrate that delayed administration with GTS-21, a selective α7nAChR agonist that mimics vagus nerve stimulation, could achieve more effective bacterial clearance and improved survival in our model of CLP-induced bacterial peritonitis. More importantly, to our knowledge, our data illustrate for the first time that enhanced influx of monocytes from the spleen into the infected peritoneal cavity and increased functional activity of these recruited monocytes within the peritoneum accounts for the enhanced anti-infectious immunity upon selective α7nAChR activation. Further basic and clinical researches are needed to provide mechanistic insight into the neuroimmune modulation and may pave an avenue for potential treatment strategies for patients suffering from sepsis or other infectious diseases.

## Data Availability Statement

The raw data supporting the conclusions of this article will be made available by the authors, without undue reservation.

## Ethics Statement

The animal study was reviewed and approved by the Scientific Investigation Board of Tianjin Medical University.

## Author Contributions

TM planned and designed experiments. J-NH, YL, S-CL, TZ, G-BC, and JZ performed experiments. TM, J-NH, and YL reviewed data and wrote the manuscript. J-NH and YL contributed equally. All authors contributed to the article and approved the submitted version.

## Funding

Research reported in this publication was supported by the National Natural Science Foundation of China (grants 82172122, 81871546, and 81471841 to TM), Tianjin Key Medical Discipline (Specialty) Construction Project, and the Tianjin Research Innovation Project for Postgraduate Students (No. 2020YJSB188 to J-NH).

## Conflict of Interest

The authors declare that the research was conducted in the absence of any commercial or financial relationships that could be construed as a potential conflict of interest.

## Publisher’s Note

All claims expressed in this article are solely those of the authors and do not necessarily represent those of their affiliated organizations, or those of the publisher, the editors and the reviewers. Any product that may be evaluated in this article, or claim that may be made by its manufacturer, is not guaranteed or endorsed by the publisher.

## Supplementary Material

Supplementary Figure 1 Representative dot plot showing the SSC x FSC gate of the sample from GTS-21 and vehicle treated mice in the cytometry analyzes.

Supplementary Figure 2 Representative histogram of Ly6C- and Ly6C+ of the sample from GTS-21 and vehicle treated mice in the cytometry analyzes.

Supplementary Figure 3 Representative dot plot showing the SSC x FSC gate of the sample from splenectomized mice treated with GTS-21 and vehicle in the cytometry analyzes.

Supplementary Figure 4 Representative histogram of Ly6C- and Ly6C+ of the sample from GTS-21 and vehicle treated splenectomized mice.
