# Supplementary figures and images for: The α7 Nicotinic Acetylcholine Receptor Agonist GTS-21 Improves Bacterial Clearance via Regulation of Monocyte Recruitment and Activity in Polymicrobial Septic Peritonitis
Source: Front Immunol. 2022 Mar 4;13:839290. doi: 10.3389/fimmu.2022.839290 (PMC8931331; doi:10.3389/fimmu.2022.839290)

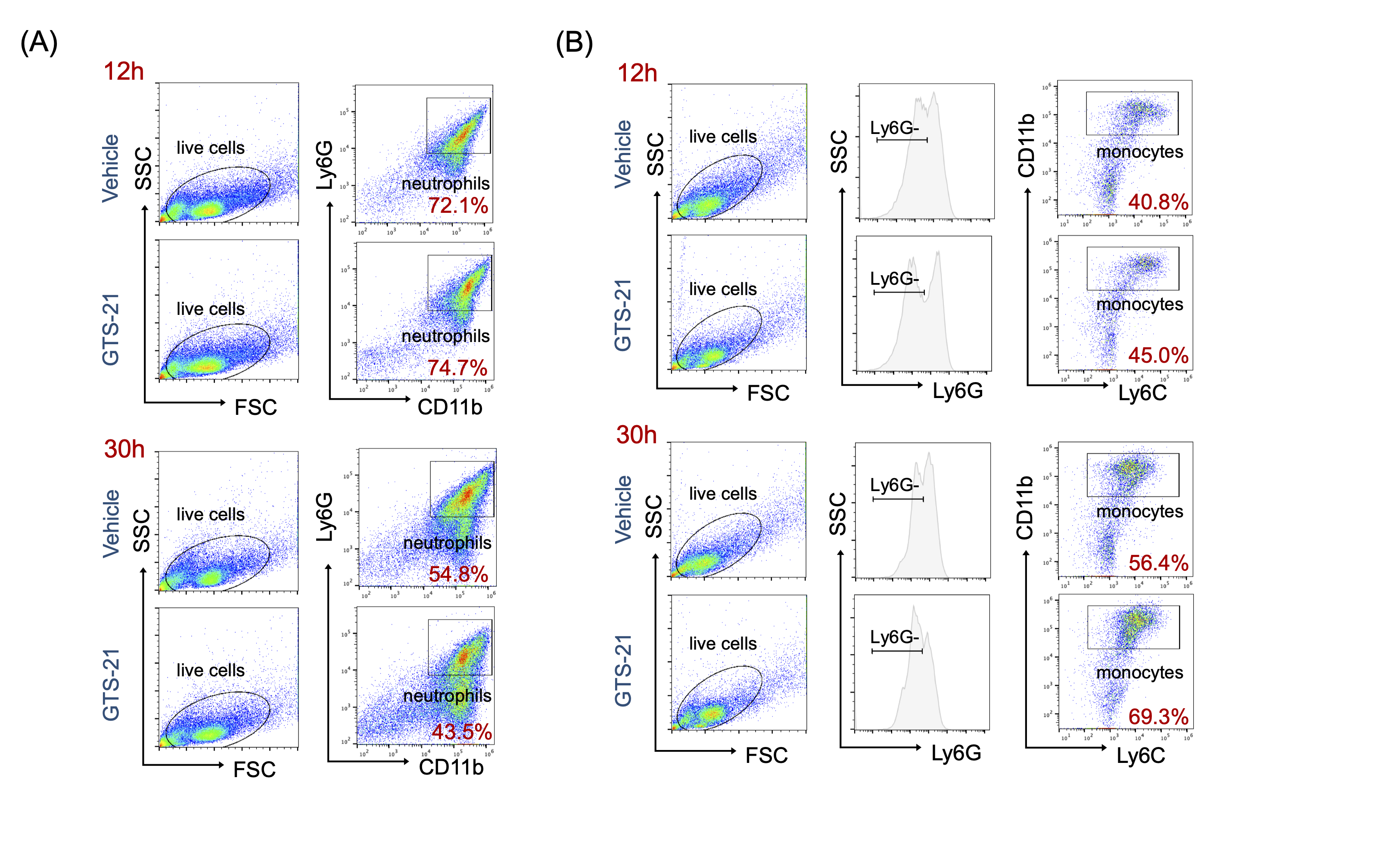

Supplement: Supplementary Figure 1 — Representative dot plot showing the SSC x FSC gate of the sample from GTS-21 and vehicle treated mice in the cytometry analyzes. [file Image_1.tiff]

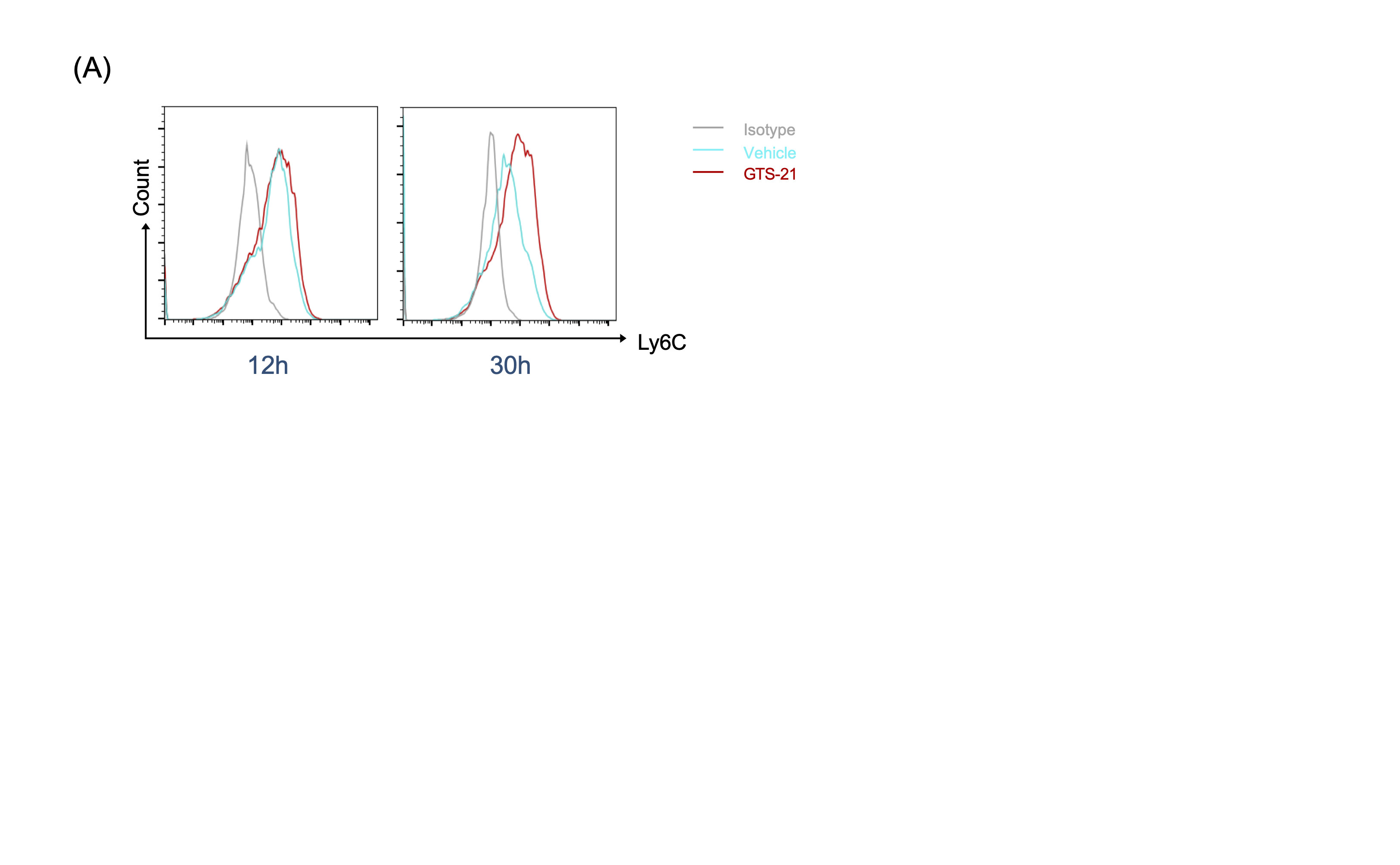

Supplement: Supplementary Figure 2 — Representative histogram of Ly6C- and Ly6C+ of the sample from GTS-21 and vehicle treated mice in the cytometry analyzes. [file Image_2.tiff]

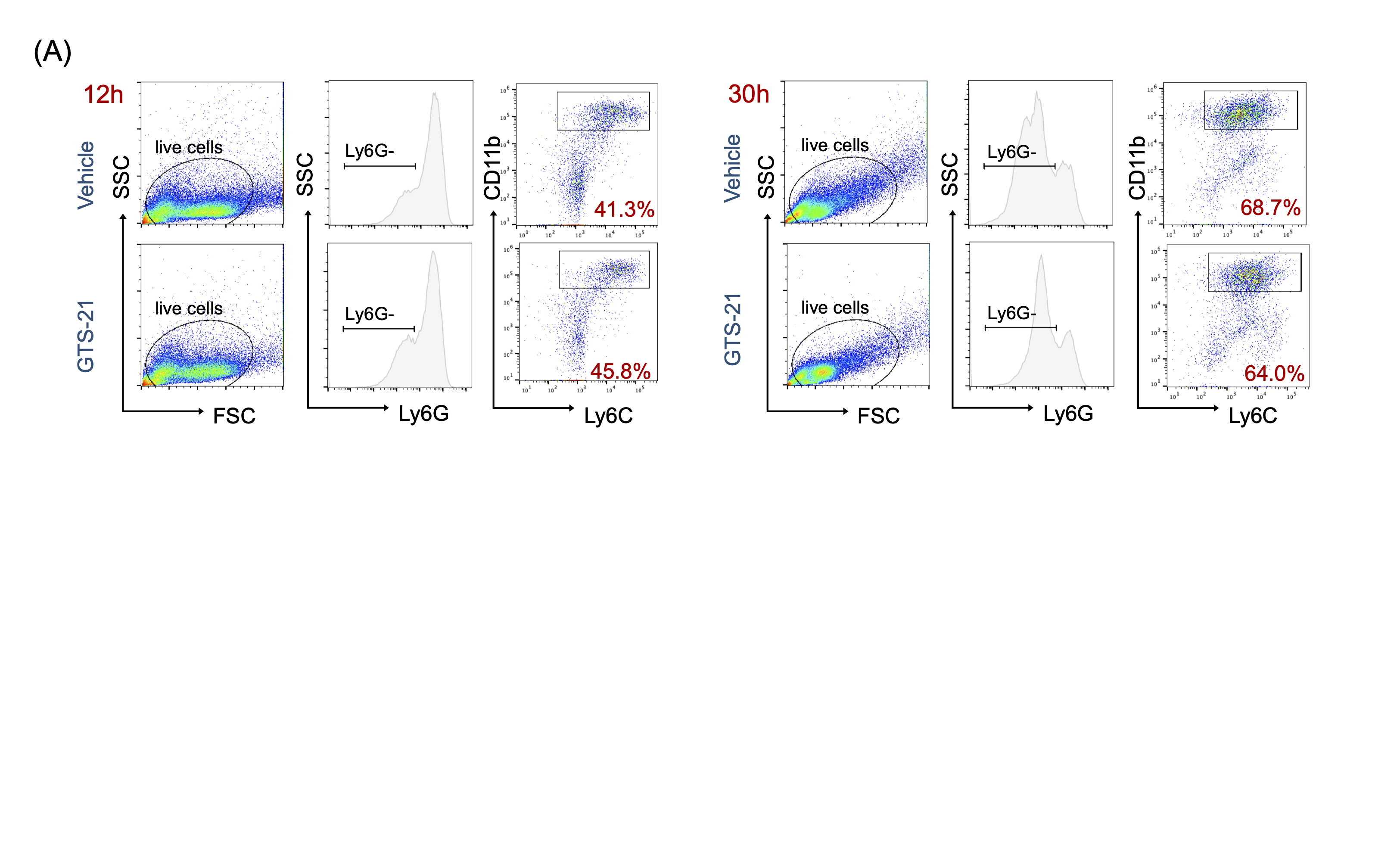

Supplement: Supplementary Figure 3 — Representative dot plot showing the SSC x FSC gate of the sample from splenectomized mice treated with GTS-21 and vehicle in the cytometry analyzes. [file Image_3.tiff]

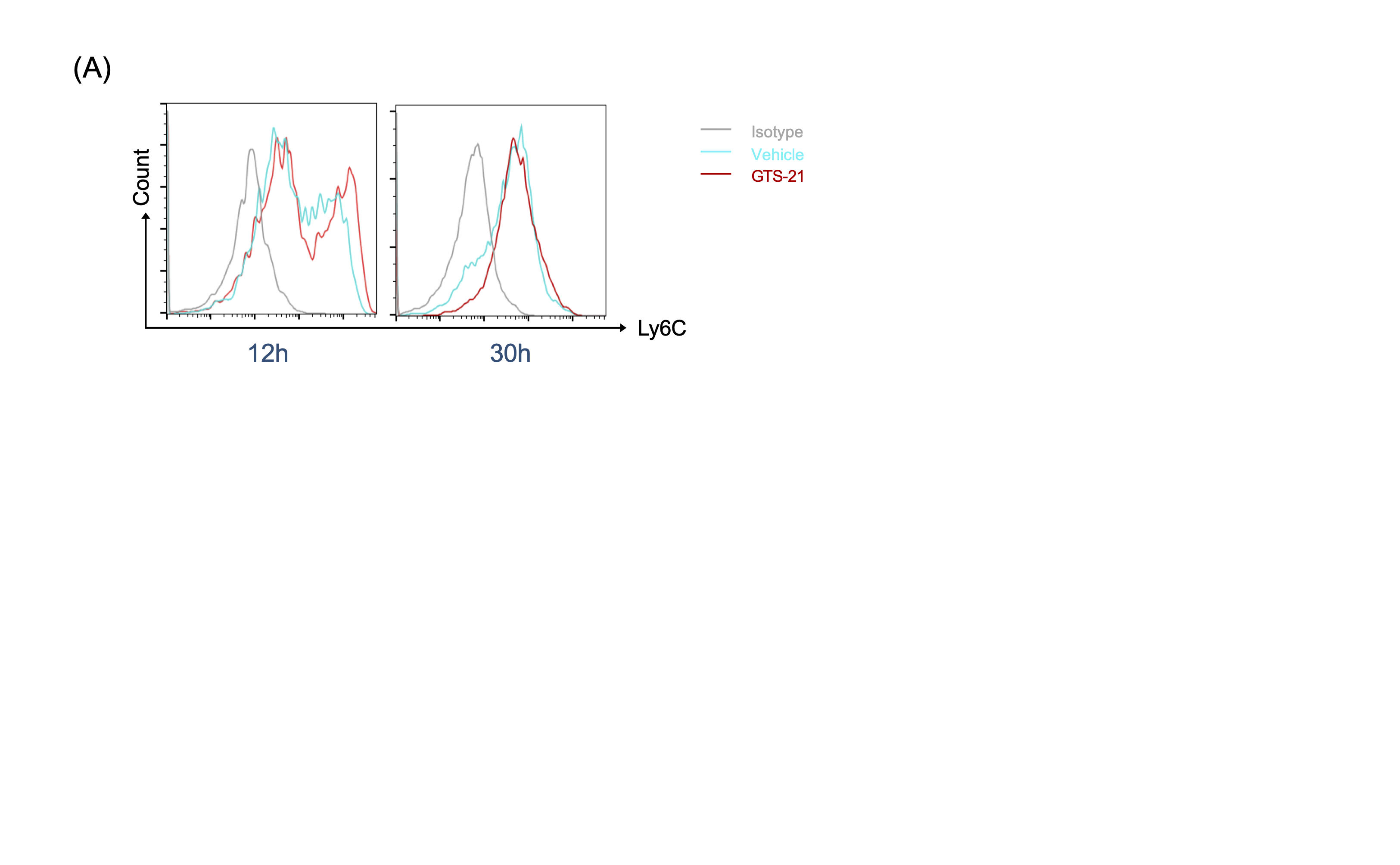

Supplement: Supplementary Figure 4 — Representative histogram of Ly6C- and Ly6C+ of the sample from GTS-21 and vehicle treated splenectomized mice. [file Image_4.tiff]
